# Supplementary material for: Are carbon emissions trading and green financial instruments synergistic? -Comprehensive quantitative research based on content analysis
Source: PLoS One. 2024 Mar 7;19(3):e0298601. doi: 10.1371/journal.pone.0298601 (PMC10919601; doi:10.1371/journal.pone.0298601)
Supplement: S1 Data — (ZIP) [file pone.0298601.s001.zip › Data/地级市Z_F工作报告.zip!/║╙─╧/╓ú╓▌╩╨/└┤╫╘╣1⁄2╓┌║┼í╛┬φ┐╦╩2╛▌═°í┐_2015.docx]

各位代表：

现在，我代表市人民政府向大会报告工作，请予审议，并请各位政协委员和列席人员提出意见。

一、2014年工作回顾

2014年，是新一届市人民政府履职的第一年。面对错综复杂的国内外形势，我们在省委、省政府和市委的正确领导下，深入贯彻党的十八大、十八届三中四中全会和习近平总书记系列重要讲话精神，紧紧围绕“三大一中”战略定位，抓改革、强投资、调结构、求提升，较好完成了市十四届人大一次会议确定的各项任务，郑州都市区建设迈出新的步伐。

（一）千方百计稳增长，经济社会持续健康稳步发展。在经济下行压力持续加大的严峻形势下，我们扭住发展不放松，着力以强投资为稳增长奠定基础、以稳增长为调结构创造条件、以调结构为稳增长提供支撑，经济运行呈现出“稳中有进、稳中趋优、稳中向好”的发展态势。初步统计，全市地区生产总值完成6783亿元，增长9.5%；规模以上工业增加值3094亿元，增长11.2%；地方公共财政预算收入833.9亿元，增长15.2%；固定资产投资5259.6亿元，增长20.1%；社会消费品零售总额2913.6亿元，增长12.7%；城镇居民人均可支配收入29095元，增长9.8%；农民人均纯收入15600元，增长11.4%。主要经济指标在全国35个大中城市中位次持续前移，经济发展的协调性不断增强。

（二）强力推进求突破，航空港实验区建设全面提速。坚持把实验区建设放在全市工作首位，推动枢纽建设、产业培育、体制机制创新等重点领域发展，实验区开发建设取得重大进展。大枢纽建设全面推进，机场二期主体工程提前封顶，外围城际铁路、高速公路等重大工程加快建设；机场客运量达到1580万人次，货邮吞吐量37万吨，增速均居全国大型机场首位。大产业培育初见成效，手机产量突破1.4亿部，全球重要的智能终端生产基地正在形成；菜鸟骨干网、友嘉产业园等一批重大项目入驻建设；中部国际电子商务产业园建成运营；成功举办首届郑州航展，穆尼飞机“郑州一号”下线交付，我市成为中西部地区首个生产通航认证飞机的城市。体制机制创新不断深化，实施市级行政执法权向实验区全面委托，复制实施上海自贸区创新制度9项，智能终端出口退税资金池投入运行，中国郑州航空港引智试验区获批。实验区影响力在扩大、吸引力在增强，已成为我省对外开放的重要平台。

（三）突出重点抓改革，发展动力和市场活力持续增强。坚持问题导向、市场导向、需求导向，重点抓好第一批58项重点改革事项，一些改革走在了全省前列。着眼于简政放权、提高行政效率，加快政府职能转变，完成本轮政府机构改革；持续深化行政审批制度改革，积极推进“两集中两到位”改革，形成了“一窗式”受理、“一站式”服务、“一条龙”审批的行政审批新模式；在全省我们率先启动“五单一网”制度改革；目前40个市级审批职能部门共受理审批事项131.5万件，全部在规定的1、3、7个工作日内办结，行政审批效率持续提升。着眼于激发市场主体活力，持续深化金融支持小微企业体制机制改革，在全国率先打造金融支持小微企业服务体系，在全国首设小微企业创业投资基金，29家科技型小微企业在“新三板”挂牌上市，居中部六省首位；以“共保体”模式支持科技型企业发展，小微企业贷款余额1850亿元、增长36%；还深化了工商登记制度改革，市场主体、注册资金分别增长了83%、255%。着眼于提高政府性资源配置能力，持续深化土地管理制度改革，实行国有经营性用地使用权网上交易招拍挂，实现了土地交易充分市场化和政府收益最大化，真正建立起了棚户区改造政府主导拆迁安置、市场化运作的新机制；开展闲置建设用地集中清理处置专项行动，累计盘活土地12.4万亩，近年来供地率首次突破60%，扭转了多年来供地率低的局面；持续深化投融资体制改革，健全完善“立融用管还”的全产业链发展模式，市8家投融资公司融资240亿元，是前3年融资总和的2.5倍；在全国率先发行首只12亿元“市政债”。探索建立了产业发展基金和股权投资资金，有效地撬动了社会资金扶持战略支撑产业发展。还稳步推进了财税体制改革、事业单位分类改革、保障性住房“三房合一”改革、市属国有企业改革、文化体制改革、农村综合改革等工作，改革红利不断得到释放。

（四）以人为本促提升，新型城镇化建设提质增速。以人的城镇化为核心，科学推进新型城镇化，城镇化率提高到68.2%。坚持规划引领，进一步完善都市区规划体系，完成“三规划一设计”编制。加快畅通郑州工程建设和公交都市建设，城市轨道交通、“井字+环线”快速路网、市域快速通道、高速公路、国省干线公路和县域路网等工程强力推进，10条市域快速通道9条完工，16座新增环城互通式立交10座建成，17个环城高速互通式立交建成投用，三环快速化、黄河路下穿北编组站隧道等重点工程建成通车，累计打通城市断头路128条，市区新增公共停车泊位2.93万个。积极配合推进“米”字形高铁网建设，高铁南站完成规划选址，郑开城际铁路通车运营。启动 “六旧九新”片区改造项目106个、十字景观大道和中央商务区建设项目158个，完成市场外迁46家，加快推进“引热入郑”工程，市区新增供热能力1630万平方米，市区新建成污水处理厂3家、新增污水日处理能力45万吨，一批水电气暖等公共服务设施加快建设或建成投用。启动大棚户区拆迁改造项目326个，开工建设安置房21.4万套，实施历史文化和自然生态风貌特色村保护提升工程。以智慧城市建设为载体，扎实开展城乡环境综合整治和城区交通秩序综合整治，顺利通过国家卫生城市复审。

（五）多措并举调结构，现代产业体系加快构建。坚持扩大优质增量与调整优化存量并举，加快构建现代产业体系，推动经济转型迈出坚实步伐。工业七大主导产业增加值完成2160亿元，增长13.3%，对全市工业经济增长的贡献率达到82.7%。四大战略性产业比重提高到46.5%，高耗能行业比重下降到42.3%，战略性新兴产业比重首次超过高耗能产业，标志着我市工业结构调整取得重大突破。“两化”融合深入推进，宽带中国示范城市获批，国家级互联网骨干直联点开通运营，河南联通中原数据基地与中国移动河南郑州数据中心建设进展顺利。高成长性服务业、生产性服务业加快发展，服务业增加值完成2890亿元。现代商贸物流、文化创意旅游业提质增速，金融集聚核心功能区建设取得新进展，郑商所国际期货定价中心地位进一步提升。电子商务发展迅猛，交易额达到2800亿元，增长30%以上。房地产业持续平稳健康发展。产业集聚效应进一步显现，产业集聚区对工业增长、投资拉动、就业创业的贡献率分别达到78%、60%和55%。都市生态农业加快发展，粮食生产保持稳定，农业组织化、机械化、产业化、科技化水平稳步提升，市级以上农业产业化集群达26个，农产品加工业收入达1200亿元，农产品质量监测合格率居全国前列。

（六）双轮驱动强支撑，城市综合竞争力明显提升。深入实施了开放创新双驱动战略，对外开放水平和自主创新能力不断增强。对外开放取得突出成效。我们抢抓国家 “一带一路”战略机遇，强力推进航空港、国际陆港、郑欧班列、跨境贸易电子商务服务试点、各类海关特殊监管区域和综合性大口岸等政府性要素平台体系建设，我市成为丝绸之路经济带重要节点城市。郑欧班列常态化运营，班次密度、货重货值均居中欧班列首位。国际陆港累计实现集装箱吞吐量12.5万标准箱。跨境贸易电子商务服务试点加快推进，位居全国同类试点城市前列。汽车整车进口口岸正式运行，成为全国首家陆港型汽车口岸。肉类口岸、食品药品口岸、粮食口岸加快建设。深入开展集群招商和产业链招商，签约总额3124亿元，实际利用外资35.4亿美元、增长6.5%。科技创新步伐明显加快。围绕主导产业布局“创新链”、围绕创新资源谋划“产业链”、围绕科技创新项目建立“服务保障链”、围绕“两金一扶”完善“资金链”、围绕人才培育构建“技术链”，着力推进“两器一园”创新创业综合体建设，健全“产学研政资介”相结合的科技自主创新体系。高新技术产业产值完成5600亿元、增长22%，科技进步对经济增长的贡献率达到59%。

（七）统筹发展惠民生，各项社会事业协调发展。坚持财政向民生领域倾斜，民生支出658.8亿元，占预算支出的71.7%。积极推进就业创业，发放小额担保贷款10亿元，新增城镇就业15.3万人，农村劳动力转移就业11.4万人。优化教育资源配置，新建幼儿园60所、新增学位1.94万个，市区新建改扩建中小学校34所、新增学位4.86万个，5.56万进城务工人员随迁子女实现“应入尽入”。加强社会保障，城乡居民基本养老金、企业退休人员基本养老待遇、城乡低保对象等生活困难群众保障标准进一步提高，新建“儿童之家”100个，新建农村示范性养老服务中心90所，我市蝉联全国“七星级慈善城市”。 公共卫生服务均等化水平显著提高，片医特色基层卫生服务体系日趋完善，新增床位3362张。全面落实“单独二孩”生育政策，人口自然增长率控制在5.7‰以内。深入实施文化惠民工程，基层公共文化设施和公共文化服务体系不断完善，大运河通济渠郑州段列入《世界遗产名录》，成功举办黄帝故里拜祖大典、国际少林武术节、世界旅游城市市长论坛等重大活动。加强生态环境保护，编制实施《美丽郑州规划》，市区拆改燃煤锅炉88台，三环内80%以上工业企业完成外迁，淘汰黄标车和老旧车辆4.88万辆；对渣土车行业进行了整合，加强了渣土车管理和市区建筑工地扬尘治理，取得了初步成效；工业企业脱硫脱硝治理力度不断加大，主要污染物排放总量完成省定控制目标。全国水生态文明试点城市建设加快推进，重点河道拦蓄水、重点水系生态修复提升和引水入密等工程相继开工，南水北调主干渠按期通水。植树造林8.5万亩，建设生态廊道880公里，市区新增绿地1620万平方米，成功申办第11届国家园博会，荣获国家森林城市称号。农业农村基础设施建设不断加强，投资1.4亿元解决了28.3万人农村饮水安全，对124座病险水库进行了除险加固。完成易地扶贫搬迁2.7万人、整村推进25个，实现脱贫4.47万人。

（八）转变作风提效能，政府自身建设不断加强。全面贯彻落实中央八项规定和省委、市委有关规定，围绕反对“四风”，扎实开展党的群众路线教育实践活动，政风建设取得新的成效，全市会议费同比下降57.5%，“三公”经费同比下降14.6%。坚持依法行政，制定修订政府规章9件、清理112件，提请市人大审议法规草案4部，办理行政复议案件1218件。自觉接受人大监督、政协监督和社会监督，办理省人大建议19件、省政协提案41件、市人大议案建议619件、市政协提案590件，议案提案办结率和见面率均为100%、满意率99%。深化提升网格化管理，狠抓安全生产、食品药品监管，扎实推进平安郑州建设，社会大局保持和谐稳定。

加强社会主义核心价值观教育和精神文明建设，全国文明城市复创有望成功。国防教育、国防后备力量建设和“双拥”共建取得新成效。外事、侨务、对台、民族、宗教、统计、审计、档案、史志、气象、地震、人防、社科等工作，都取得了新的成绩。

2014年也是郑州都市区建设三年行动计划的收官之年。三年来，我们紧紧围绕“三大主体”工作，抢抓中原经济区和航空港实验区建设机遇，凝心聚力，克难攻坚，干成了一系列具有奠基性、战略性、标志性的大事要事，郑州发展的思路更加明晰，现代产业体系培育步伐明显加快，城乡基础设施建设力度明显加大，对外开放层次和水平明显提高，政府适应市场化要求的运作机制和水平不断提升，经济社会发展呈现了好的趋势、好的态势、好的气势。与2011年相比，地区生产总值净增了1820亿元，地方公共财政预算收入净增了332亿元，固定资产投资净增了2380亿元，经济份额占全省的比重不断提高，在连续三年宏观经济下行压力持续加大的情况下，为全省的发展作出了积极的贡献。郑州率先全面建成小康社会、加快现代化建设的基础更加坚实，支撑更加有力，前景更加广阔！

各位代表！我市经济社会发展取得的成绩，是省委、省政府和市委正确领导的结果，是市人大、市政协和社会各界大力支持的结果，是全市人民团结奋斗的结果。在此，我代表市人民政府，向辛勤工作在各行各业的广大干部群众，向驻郑人民解放军、武警官兵、公安干警以及社会各界人士，向关心支持郑州发展的港澳台同胞、海外侨胞和朋友们，表示崇高的敬意和衷心的感谢！

成绩面前，我们也清醒地认识到存在的问题和不足，主要是：经济下行压力较大，传统行业生产经营普遍困难；结构不合理问题尚未实现根本性转变，资源型工业比重仍然较大，新兴产业和现代服务业比重还不高，自主创新能力还不强，都市农业与二三产业融合度不够；城市承载能力依然不足，资源环境约束加剧，交通拥堵、大气污染等问题依然突出；食品药品监管、安全生产还存在薄弱环节，政府公共服务能力和水平与群众期盼还有不小差距等。对于这些问题，我们将采取措施，不断加以改进和解决。

二、2015年工作总体要求和主要目标

当前，我市经济和全国、全省一样，已进入“增速换挡、结构优化、动力转换”的经济发展新常态，但是，总体上仍处于发展的重要战略机遇期。中原经济区建设特别是郑州航空港经济综合实验区建设，郑州成为国家丝绸之路经济带重要节点城市，这些都为郑州带来了重大发展机遇。我市的区位优势，特别是立体综合交通枢纽建设步伐的加快，多式联运体系的构建、物流集疏能力的提升，都将进一步增强郑州承接产业转移的比较优势。特别是近年来我市以航空港、国际陆港、郑欧班列、跨境贸易电子商务服务试点为载体，着力打造开放平台，营造国际化营商环境，在内陆地区形成了对外开放的新的比较优势。我市的综合优势不断在增强，机遇仍然大于挑战。我们必须坚定信心，乘势而上，加快经济转型，推动科学发展。

2015年是全面深化改革的关键之年，是全面推进依法治国的开局之年，也是全面完成“十二五”规划的收官之年。今年政府工作的总体要求是：深入贯彻中央、省委、市委各项决策部署，紧紧围绕打造大枢纽、发展大物流、培育大产业、建设以国际商都为特征的国家中心城市的“三大一中”战略定位，坚持稳中求进总基调，以提高经济发展质量和效益为中心，以“抓改革创新、强投资开放、促结构转型、求民生改善”为总要求，抢抓机遇，主动适应经济发展新常态，提升“三大主体”工作，全面推进依法治市和以航空港实验区为统揽的郑州都市区建设，促进经济平稳健康发展和社会和谐稳定。

今年经济社会发展的主要预期目标是：在优化结构、提高质量、改善环境的基础上，地区生产总值增长9%左右；规模以上工业增加值增长10%；固定资产投资增长18%；地方公共财政预算收入增长13%；社会消费品零售总额增长12%；进出口总额增长5%；实际利用外资与去年大体持平；城乡居民收入增长与经济增长同步；节能减排降碳完成省下达目标。

实现上述要求和目标，我们将紧紧围绕总要求，在保持经济运行总体平稳的同时，突出抓好五大重点任务。一是突出抓好深化改革。以航空港实验区体制机制创新、“五单一网”改革、行政审批制度改革、投融资体制机制改革等重点领域改革带动改革全面深化，不断增强区域发展的活力和动力。二是突出抓好开放创新双驱动。以跨境贸易电子商务和期货交易为引领，以综合交通信息枢纽和各类口岸等政府性要素平台为支撑，以空港、陆港、各类海关特殊监管区域等为载体，着力打造内陆对外开放高地；以深化提升人才引进培育机制为引领，以健全完善“两金一扶”政策环境为支撑，以加快“两器一园”创新创业综合体建设为载体，着力构建科技自主创新体系。三是突出抓好现代产业体系构建。围绕打造“万亿级电子信息产业基地、5千亿级汽车及装备制造基地和6个千亿级产业基地”，大力发展先进制造业和高成长服务业，加快构建大都市战略支撑产业体系。四是突出抓好新型城镇化建设。以人的城镇化为核心，以大棚户区改造、城市综合承载能力提升、畅通郑州工程为重点，以城市精细化管理先行区建设为带动，构建“都市核心区、外围组团、新市镇、新型农村社区”的四级现代城镇体系，着力打造自然之美、社会公正、城乡和谐的现代田园城市。五是突出抓好民生改善。围绕“大气污染、交通拥堵、扶贫开发、社会保障、平安稳定”等与群众生产生活紧密相关的突出问题，进一步加大工作力度，提高公共服务水平，使改革发展成果更多、更好、更公平地惠及广大人民群众。

各位代表！我们要不断认识新常态、努力适应新常态、积极作为新常态，就要紧紧扭住发展不放松，狠抓改革深化，狠抓开放创新，狠抓转型发展，狠抓民生改善，努力在新常态下迈上新台阶！

三、2015年重点工作

今年，我们将重点做好以下工作：

（一）坚持“三大一中”战略定位，着力开创郑州都市区建设新局面。牢牢把握“一带一路”重大机遇，全面落实《河南省全面建成小康社会加快现代化建设战略纲要》，围绕“建成‘连通境内外、辐射东中西’的国际综合交通信息枢纽，形成‘买全球、卖全球’的国际物流网络，构建以开放经济和智慧经济为统领、先进制造业和高成长服务业为主体的现代产业体系，塑造自然之美、社会公正、城乡和谐的现代田园城市”的目标，谋划推进中国郑州自贸区，全力推动国际商都规划建设，同步推进编制“十三五”规划，力争郑州自贸区获批，使我市成为中西部地区率先获批内陆型自贸区的城市。我们将以国际商都规划为指导，加快推进实施都市区新三年行动计划，力争用三年时间初步形成国际化现代化立体综合交通枢纽，初步形成大都市战略支撑产业体系，初步形成城乡一体化发展格局，初步形成重要的内陆开放高地，初步形成可持续发展的生态环境基础，初步形成社会治理现代化体系，在全省率先全面建成小康社会，为建设国际商都奠定基础。

（二）坚持先行先试，着力实现航空港实验区建设大突破。全面实施航空港实验区建设体制机制创新示范区总体方案，加快构建既符合国际惯例又适应实验区发展需要的开放型经济新体制新机制。全力打造现代综合交通枢纽，机场二期建成投用，配合推进“米”字形高铁网建设，加快推进高铁南站项目建设，郑州东站至机场城际铁路、机场高速改扩建等快速通道建成通车，大力拓展“空海铁公”多式联运业务，持续提升郑州现代综合交通枢纽的集疏能力。积极构建航空港产业体系，加快全球重要的智能终端生产基地建设，全面推进与富士康的战略合作专案，正威、酷派、天语、中兴等项目开工或建成投产，手机检测研究院等支撑平台初成体系，力争智能手机产量突破2亿部。以IBM郑州数据中心等10大招商项目、友嘉产业园等10大产业项目、河南电子口岸服务中心等10大要素平台项目为重点，加大产业培育引进力度，力争签约总额突破1500亿元。办好2015年郑州航展，支持穆尼飞机组装生产、通航机场布局建设等通航产业发展，争取列入国家首批低空空域开放试点区域。

（三）坚持深化改革，着力增强发展动力。深化行政审批制度改革。全力推进政府“五单一网”制度改革，3月底“五个清单”向社会公布，年底前“四级联动”的政务服务网全面运行；持续深化“两集中两到位”改革，进一步提升行政审批效率；完成政府机构改革任务和公务用车制度改革。深化投融资体制改革。着力增强政府投融资公司融资能力，在推广PPP模式、拓展融资渠道、做活多元业务上求突破；完善产业发展基金和股权投资资金管理公司与商业银行、风险投资机构互惠共赢新模式，争取产业发展引导基金规模市本级突破100亿元。深化农村综合改革。创新农业投融资机制，推进农村产权制度改革，按照要求完成农村土地承包经营权确权登记颁证工作，积极谋划做好永久性基本农田调整划定工作。持续深化土地管理制度改革、金融支持小微企业改革、财税体制改革、文化体制改革、医疗卫生体制改革、国有企业改革、集体林权制度改革、社会保险“五险合一”改革等重点领域改革。

（四）坚持以人为本，着力推进新型城镇化。坚持“一基本、两牵动、三保障”，加快推进新型城镇化。加快农业转移人口市民化。围绕产业合理布局、人口合理分布，有序推进户籍制度改革，统筹推进附着在户籍上的教育、医疗、就业、社保等领域的配套改革，加快推进基本公共服务均等化，积极开展农业转移人口市民化成本分担机制探索。加快大棚户区改造。着力推进大棚户区改造项目和“四个优先”区域内村庄拆迁改造，力争年底市辖区内村庄拆迁基本完成；加快安置房建设，力争年内50%的动迁群众实现回迁。加快畅通郑州工程建设。开工建设轨道交通3号线一期，加快建设5号线、1号线二期，建成2号线一期、南四环至郑州南站城郊铁路的主线及全部车站主体。加快推进“井字+环线”快速路工程，开工建设农业路快速通道、南三环北三环东延高架等工程，未来路、经三路下穿金水路隧道和陇海快速路等工程竣工通车；238条支线路网工程年底前完工；加快推进中原西路等有轨电车示范线建设；继续推进公交客运场站、快速公交迁建等项目建设。完善城市基础设施。强力推进坚强智能电网建设；进一步提升供水保障能力，完成柿园、白庙水厂水源置换，让全市人民尽快饮用南水北调优质水；加快建设双桥污水处理厂，建成运行郑州新区污水处理厂；开工建设郑州东部垃圾焚烧发电厂，加快推进大型压缩式垃圾转运站和航空港区垃圾处理场建设，西部和中部餐厨垃圾处理厂年内建成投运；新建改建自来水管道120公里、供热管网80公里，新增供热能力1900万平方米。提升城市精细化管理服务水平。以筹办国家园博会为契机，以智慧城市建设为载体，把铁路编组站以东、金水河以北、中州大道以西、北三环以南约40平方公里的区域作为城市精细化管理服务先行区，构建以三级三类便民服务中心为依托的全方位、多层次的公共服务体系，全面提升以道路为载体的水电气暖的保障水平，加快打造15分钟生活圈，切实让群众享受到城市建设成果。

（五）坚持结构调整，着力构建现代产业体系。加快工业结构优化升级。做大做强战略支撑产业，以富士康、海尔等企业为龙头，加快打造全国领先的电子信息产业制造基地，力争产值达到3500亿元；依托宇通客车、东风日产、郑煤机、中铁盾构等企业，着力打造世界级客车生产基地、全国先进装备制造业基地，力争汽车及装备制造业产值达到2600亿元。培育壮大战略性新兴产业，巩固提升超硬材料、新型耐材等产业优势，加快培育物联网、云计算、大数据、3D打印、智能机器人等新兴业态，力争产值突破2000亿元。改造提升传统优势产业，着力推进现代食品制造、铝业精深加工、品牌服装及家居制造集群化、品牌化、高端化发展，力争产值突破3000亿元。加快产业集聚区发展，力争完成固定资产投资2400亿元。积极推动“两化”深度融合，大力实施“智能制造”工程，加快信息消费试点城市建设。加快推进服务业提质增效。以服务业综合改革试点工作为抓手，促进电子商务和现代物流融合发展。大力发展航空物流，着眼培育现代物流集疏体系，形成航线网络，全年航空货运量达到50万吨。按照“三多、三并举、一突出”的要求，加快推进电子商务发展，积极支持唯品会、聚美优品、京东商城等电商平台发展，力争电子商务交易额达到3500亿元。积极推动商贸业转型升级，完成剩余55家市场外迁任务，大力促进市场外迁承接地产业集聚，支持华南城、金源百荣商贸城等项目加快建设；加快商务中心区和特色商业街区建设，力争二七核心商圈销售收入达到1000亿元、郑东新区核心商圈销售收入达到300亿元。大力发展金融业，拓展金融服务领域，拉长金融产业链条，积极发展直接融资，提高郑东新区金融核心功能集聚区存贷款规模占全省的比重，提高直接融资在全社会融资中的比重，努力打造郑东新区国际化区域金融中心。大力发展文化创意旅游业，深度挖掘黄河文化、黄帝文化、少林文化、商都文化内涵，加快发展全域旅游，积极打造国家旅游集散中心；加快建设中牟绿博文化产业园，着力打造国际化时尚文化创意旅游中心；加快建设中原动漫文化创意中心，促进动漫产业优化升级。大力发展会展业，加快打造国家区域性会展中心。着眼于商业模式创新和消费结构升级，大力发展健康养生、养老服务、通用航空、信息消费等高成长性服务业。加快申报国家服务外包示范城市，争创国家级养老服务业综合改革试点。加强对商品房、安置房、保障房相关政策研究，优化土地和房地产市场供应结构，促进房地产业平稳健康发展。加快发展都市生态农业。全面落实国家“三农”政策，加强农田基础设施建设，促进粮食增产、农业增效、农民增收；促进一产与二产、三产融合发展，新建高标准农田2.8万亩、可追溯标准化“菜篮子”生产基地4万亩、现代都市生态农业示范园6万亩，市级以上农业产业化重点龙头企业达到429家，完成生产环节农畜产品质量安全追溯体系建设。加快重点项目建设。力争在郑省重点项目的数量和投资规模占全省的比重达到50%以上、省市重点项目一季度立项审批和项目开工达到全年任务的80%以上、完成投资3600亿元以上。

（六）坚持开放创新双驱动，着力构建国际化现代化开放型经济体系和科技创新体系。打造开放平台。加快推进新郑综合保税区业务拓展、郑州特种商品进口指定口岸和经开综合保税区申建工作，加快出口加工区B区建设、确保7月封关运行；推进通关通检机制创新，加快打造全国邮政第四转运口岸，力争跨境贸易电子商务服务试点提质增量、郑欧班列开行150班次以上；建成运营进口肉类指定口岸，加快汽车口岸二期和进口粮食、食品药品等指定口岸建设；着力推进以电子口岸为载体的 “大通关”体系建设，构建“一站式”通关服务平台。拓宽开放领域。继续复制放大上海自贸区成功经验，向社会资本推介一批服务业、社会事业和基础设施等领域的重大项目，大力发展服务贸易，推动加工贸易扩大规模、提高质量，鼓励优势企业开展国际合作。积极开展大招商。大力引进“三力型”项目，配合办好河南投洽会、黄帝故里拜祖大典等重大活动，力争全年招商引资达到1800亿元。构建科技创新载体平台。加快构建以国家专利审查协作河南中心、国家技术转移郑州中心、中科院过程所郑州分所等为重点的创新平台；全力推进20个创新创业综合体建设；围绕主导产业和新兴产业发展，着力打造3个产业技术创新研究院；扶持企业建立研发中心，力争新建市级研发中心100家、省级以上20家，认定行业技术领域院士工作站10家。强化自主创新能力。大力培育科技型企业，规划建设科技服务业集聚区，谋划建设郑州自主创新示范区；以“两器一园”为载体，加快科技成果转化；鼓励企业商务模式创新，构建线上线下相统一的全渠道销售模式和个性化、定制化生产模式。加快人才培育引进。全面启动“智汇郑州·1125聚才计划”，实现人才链、创新链、产业链和服务链的融合对接，推动人才集聚，为都市区建设提供人才智力支撑。

（七）坚持改善生态环境，着力打造美丽郑州。以生态文明先行示范区建设为载体，坚持源头严防、过程严管、后果严惩，建设天蓝、地绿、水净的美好家园。持续推进“蓝天工程”。严格落实《郑州市大气污染防治条例》，加快高污染燃料禁燃区建设，力争市区禁燃区面积达到建成区面积的60%以上，按照计划强力推进城区燃煤锅炉拆改，淘汰社会黄标车和老旧车辆5万辆以上，城市环卫机械化清扫率达到70%以上，全市渣土清运车辆密闭改装率达到100%，城镇气化率达到92%，加快三环内工业企业外迁，做好秸秆禁烧和综合利用，强化重点行业脱硫脱硝治理，大力推进节能减排降碳工程，全面完成主要大气污染物减排任务。全面实施“碧水工程”。加快水生态文明试点城市建设，开工建设牛口峪引黄工程、石佛沉砂池向西区供水工程和环城生态水系循环等重点工程；以南水北调沿线水污染防治为重点，加强饮用水水源地环境管理，全年解决12万人农村饮水安全；开展城市河流清洁行动，对金水河、十八里河等7条城区河道进行集中整治，努力实现“水清河美”目标。大力改善城乡环境。巩固国家森林城市创建成果，积极筹办国家园博会，启动建设园博园，抓好生态廊道、综合公园、南水北调生态文化公园等建设，完成营造林13.7万亩，市区新增绿地500万平方米以上，建成区级综合性公园10个、森林体验园10个；加强爱国卫生工作，大力推进“乡村清洁工程”，加强农村环境连片综合整治和农村公路两侧“双违”整治，强化畜禽养殖企业污染治理，切实改善城乡环境。探索完善生态环境保护体制机制。建立健全生态环境补偿、环境损害鉴定评估、责任追究机制和节能减排总量预算管理机制，探索实施节能量、排污权、碳排放交易。强化环境应急能力建设，加大环境保护执法力度，让损害环境者依法付出代价！

（八）坚持为民惠民，着力保障和改善民生。全面落实政府主体责任，继续加大财政投入，认真做好省市民生“十大实事”。千方百计扩大就业，建立健全就业服务网络，加快公共就业和人才服务市场建设，加强就业创业培训，发放小额担保贷款8亿元，新增城镇就业13万人，农村劳动力转移就业8万人。促进教育均衡发展。新建城乡幼儿园40所，新增学位8000个以上；新建改扩建市区中小学30所，新增学位3万个以上；开展职业教育资源整合重组，扩大职业教育办学规模；力争中州大学升本和郑州卫校升专工作取得突破性进展。全面提升人民健康水平。持续完善片医特色的基层医疗卫生服务体系，加快推进 “一城七中心”等重大卫生项目建设，深入推进县级公立医院改革，巩固完善公共卫生和基本医疗地图式定位责任服务管理，鼓励引导社会资本举办医疗机构。落实好“单独二孩”政策，促进人口长期均衡发展。统筹城乡社会保障体系建设。坚持社会救助和保障标准与物价上涨挂钩联动机制，推进“五险合一”市级统筹信息系统建设，扩大社会保险参保覆盖面，提高城乡低保、农村五保、医疗救助补助标准以及新农合、农民大病保险筹资标准，实施机关事业单位养老保险制度改革，加强养老服务设施、救助管理设施、社会福利设施建设，做好妇女儿童工作，支持慈善事业健康发展，新开工建设保障房14.5万套、基本建成6.2万套。繁荣文化体育事业。完善公共文化服务体系，大力开展全民健身运动，抓好市民公共文化服务中心建设，力争“四中心”、“两公园”开工建设，强化文艺精品创作。实施郑州国家大遗址片区保护利用战略规划，支持登封建设华夏历史文明传承创新示范工程，推进登封世界历史文化旅游名城建设。强力推进扶贫开发。紧紧围绕“到2017年解决15.3万扶贫对象脱贫”目标，从今年起三年内投入20亿元，大力推进易地扶贫搬迁，发展旅游业等特色经济，增强贫困群众就业增收能力，在全省率先实现脱贫奔小康目标。今年完成易地扶贫搬迁4万人、整村推进20个，实现脱贫5.4万人。

（九）坚持深化提升网格化管理，着力加强社会治理体系和社会治理能力现代化建设。着眼“双安”、推进“双治”、强化“双基”，突出系统治理、依法治理、综合治理、源头治理，加快从传统社会管理向现代社会治理转变。深化提升网格化管理，以社会化服务为方向，以提升管理信息化水平为抓手，以推动工作落实为目标，进一步增强网格化管理实效。提升城乡社区管理和服务水平，增强基层自治服务功能，激发社会组织活力。加强安全生产基层基础建设，深化煤矿、非煤矿山、建筑施工、交通运输、危险化学品等重点行业和领域安全治理。建立健全监管机构，完善监管网络，强化食品药品安全监管。持续推进平安郑州建设，加大普法宣传教育力度，依法做好信访稳定工作，积极防范群体性事件和突发公共事件，严厉打击各类违法犯罪行为，努力维护社会大局和谐稳定。

（十）坚持依法行政，着力建设法治政府。以建设法治政府、服务型政府、廉洁政府为目标，以深化改革为动力，以制度约束为重点，持续推进政府系统党风廉政建设和反腐败工作，不断营造风清气正的经济社会发展环境。坚持依法行政。严格按照法定权限和程序行使权力、履行职责、推进政务公开。认真执行人大决议决定，自觉接受人大法律监督、政协民主监督、司法监督，主动接受社会公众和新闻舆论监督。研究出台重大行政决策程序规则，做到重大决策于法有据、决策与立法紧密衔接。坚持简政放权。大幅减少投资项目前置审批，取消全部非行政许可审批事项，加快建立权责清单，理清政府和市场的关系，提升政府的行政效率。坚持厉行节约。继续严格执行中央八项规定和国务院“约法三章”以及省委、市委有关规定，压缩“三公”经费，节约政府开支，降低行政成本。

加强国防动员、国防后备力量和人民防空建设，推动军民融合深度发展。支持驻郑解放军、武警部队建设，深化“双拥”共建工作。持续推进民族团结进步创建活动，促进宗教和谐和睦。全面做好统计、审计、地方志、外事侨务、对台事务、社科研究、档案管理、防震减灾等工作。

各位代表！新常态孕育新希望，新希望开启新征程。让我们紧密团结在以习近平同志为总书记的党中央周围，在省委、省政府和市委的坚强领导下，抢抓机遇，齐心协力，务实重干，克难攻坚，加快推进以航空港实验区为统揽的郑州都市区建设，为打造“四个河南”、推进“两项建设”、实现“中原更出彩”作出省会城市应有的贡献！
